# Human Respiratory Syncytial Virus Detected in Mountain Gorilla Respiratory Outbreaks

**DOI:** 10.1007/s10393-020-01506-8

**Published:** 2020-12-20

**Authors:** Jonna A. K. Mazet, Brooke N. Genovese, Laurie A. Harris, Michael Cranfield, Jean Bosco Noheri, Jean Felix Kinani, Dawn Zimmerman, Methode Bahizi, Antoine Mudakikwa, Tracey Goldstein, Kirsten V. K. Gilardi

**Affiliations:** 1 Karen C. Drayer Wildlife Health Center, One Health Institute, University of California, 1089 Veterinary Medicine Dr., Davis, CA 95616 USA; 2 Gorilla Doctors, Mountain Gorilla Veterinary Project Inc, Davis, CA USA; 3 Gorilla Doctors, Mountain Gorilla Veterinary Project Inc, Musanze, Rwanda; 4 Rwanda Development Board, Kigali, Rwanda; 5 National Zoological Park, SCBI Global Health Program, Washington, DC USA; 6 One Health Approach for Conservation, Gorilla Health, Kigali, Rwanda

**Keywords:** One health, Rwanda, Human–wildlife interface, Mountain gorillas, HRSV, Respiratory disease

## Abstract

Respiratory illness (RI) accounts for a large proportion of mortalities in mountain gorillas (*Gorilla beringei beringei*), and fatal outbreaks, including disease caused by human metapneumovirus (HMPV) infections, have heightened concern about the risk of human pathogen transmission to this endangered species, which is not only critically important to the biodiversity of its ecosystem but also to the economies of the surrounding human communities. Our goal was to conduct a molecular epidemiologic study to detect the presence of HRSV and HMPV in fecal samples from wild human-habituated free-ranging mountain gorillas in Rwanda and to evaluate the role of these viruses in RI outbreaks. Fecal samples were collected from gorillas with clinical signs of RI between June 2012 and February 2013 and tested by real-time and conventional polymerase chain reaction (PCR) assays; comparison fecal samples were obtained from gorillas without clinical signs of RI sampled during the 2010 Virunga gorilla population census. PCR assays detected HMPV and HRSV first in spiked samples; subsequently, HRSV-A, the worldwide-circulating ON1 genotype, was detected in 12 of 20 mountain gorilla fecal samples collected from gorillas with RI during outbreaks, but not in samples from animals without respiratory illness. Our findings confirmed that pathogenic human respiratory viruses are transmitted to gorillas and that they are repeatedly introduced into mountain gorilla populations from people, attesting to the need for stringent biosecurity measures for the protection of gorilla health.

## Introduction

Mountain gorillas (*Gorilla beringei beringei)* are endangered, with just 1063 individuals in two protected areas in east central Africa (Hickey et al. 2018a, 2019). Over 70% of the 604 gorillas in the Virunga Massif, which spans three national parks in Rwanda, the Democratic Republic of Congo, and Uganda, are human-habituated for visitation for observational research and tourism (Hickey et al. 2018b). The Virunga mountain gorilla population has experienced a steady growth rate, with previous studies suggesting that the human-habituated mountain gorilla subpopulation experiences annual growth while the unhabituated subpopulation does not (Kalpers et al 2003). Growth in the habituated gorilla population is likely the result of daily monitoring and veterinary intervention (Robbins et al. 2011).

The burgeoning wildlife tourism industry has increased the demand for experiences that put tourists in close proximity to wild great apes (Setchell et al. 2016). These ecotourism activities contribute to economic benefits for local people and governments by generating tourism-related employment opportunities and revenue (Homsy 1999; Nolen 2006). Despite these benefits, there is mounting evidence of pathogen transmission between humans and mountain gorillas, likely the result of their close genetic relationship and susceptibility to many of the same pathogens (Nizeyi et al. 2001, 2002; Alvarez and Vollm 2004; Cranfield 2008). Of particular concern are zoonotic respiratory pathogens because respiratory illness (RI) in mountain gorillas accounts for approximately a quarter of investigated mortalities and is the second leading cause of death after trauma (Cranfield 2008), including in infants (Hassell et al. 2017).

Morbidity from RI in mountain gorillas is high as well. A review of respiratory disease in mountain gorillas from 1990 to 2010 noted moderate to severe outbreaks of RI in twelve separate mountain gorillas groups between 2008 and 2010, in which group-wide morbidity ranged from 47 to 100% (Spelman et al. 2013). In 2009, a human virus, human metapneumovirus (HMPV), contributed to the deaths of two gorillas during an outbreak of severe RI, involving 11 of 12 individuals in a family group (Palacios et al. 2011). Further, pneumoviruses (such as HMPV) and certain rhinoviruses and coronaviruses have been identified as causative agents in morbidity and mortality events in other great ape populations, including wild human-habituated (Kaur et al. 2008; Köndgen et al. 2008; Scully et al. 2018; Patrono et al. 2018; Negrey et al. 2019) and captive (Slater et al. 2014; Szentiks et al. 2009) chimpanzees (*Pan troglodytes*), and exposure to human respiratory pathogens in captivity appears to be common in great apes, especially when human contact is frequent (Buitendijk et al. 2014; Kilbourn et al. 2003; Kooriyama et al. 2013). Another pneumovirus, human respiratory syncytial virus (HRSV), has been documented to simultaneously infect western lowland gorillas (*Gorilla gorilla gorilla*) and people in the surrounding communities in the Central African Republic (Grützmacher et al. 2016). Exacerbating the morbidity and mortality of these pneumoviruses are deadly opportunistic coinfections with *Streptococcus pneumoniae* and other bacteria (Chi et al. 2007; Grützmacher et al. 2018; Köndgen et al. 2008, 2017; Palacios et al. 2011; Unwin et al. 2013), amplifying the impact from the risk of exposure to these pathogens.

Although they are the leading causes of severe respiratory illness in children worldwide (Aslanzadeh and Tang 2011; Liu 2011), HRSV and HMPV can also cause asymptomatic infections, in which the virus is detectable in respiratory secretions in the absence of symptoms (Hall et al. 2001), and thus infected individuals may not be aware that they are infectious to others. Given the frequency of close contact with humans and the evidence for pneumoviruses contributing to RI outbreaks in habituated great apes, the potential for disease caused by repeated introductions of human pathogens into mountain gorilla populations with unknown post-infection immunity warranted further investigation (Ryan and Walsh 2011). Unfortunately, direct sampling of the respiratory tract of wild endangered gorillas for diagnostic testing is impractical except under very specific circumstances (e.g. chemical immobilization for snare removal or clinical treatment). Alternatively, non-invasive samples (samples that do not require animal handling and chemical immobilization for acquisition), such as feces, can be readily collected. Studies have shown that HMPV and HRSV can be detected in feces from lowland gorillas, bonobos (*Pan paniscus*), and chimpanzees by real-time and conventional PCR assays (Grützmacher et al. 2016, 2018; Köndgen et al. 2010).

Given the impacts of RI on mountain gorilla health and the association of human pneumoviruses with previous RI outbreaks in great apes, including mountain gorillas, our goal was to conduct a molecular epidemiologic study to determine the prevalence and impact of HRSV and HMPV infection in cases of RI in mountain gorillas from the Virunga population. Specifically, the objectives of this study were to: (1) use real-time and conventional PCR assays to detect HRSV and HMPV in fecal samples collected from wild mountain gorillas with and without signs of respiratory illness; and (2) use the findings to inform future surveillance and disease prevention efforts, such as guidelines for human visitation of mountain gorillas, to protect the health of people and gorillas.

## Materials and Methods

### Laboratory Assay Detection Limit

Real-time and conventional PCR assays to detect HMPV and HRSV (Köndgen et al. 2010) were utilized for laboratory optimization. HMPV screening was performed by real-time PCR using the NL-N 2 assay (Klemenc et al. 2012) to amplify a 170 bp portion of the conserved N (nucleocapsid) gene. Sequences for HMPV phylogenetic analysis were amplified using a conventional PCR assay amplifying a 885 bp segment of the P (phosphoprotein) gene (Piyaratna et al. 2011). HRSV screening was performed by real-time PCR (Reiche and Schweiger 2009) with a probe modification (Köndgen et al. 2010) to amplify a ~ 120 bp fragment of the conserved N (RNA-binding protein) gene. Sequences for HRSV phylogenetic analysis were amplified using two hemi-nested, conventional PCR assays specific for the two main subgroups, A and B, to amplify a 450–530 bp fragment of the G (attachment protein) gene, targeting the second hypervariable region (Sato et al. 2005).

Positive controls (PCs) included HRSV strain Long (VR-26D from ATCC, Manassas, Virginia; accession number AY911262) and HMPV strains A2 TN/94-49 (Genbank accession no.: JN184400) and B2 TN/89-515 (kindly provided by Dr. John Williams, Vanderbilt University). Analytic detection limits of the real-time PCR screening assays were determined by amplifying the N gene fragments from the HRSV and HMPV positive controls, and amplified PCR products were cloned into plasmids (Invitrogen TOPO TA Cloning Kit, Life Technologies, Carlsbad, CA), and sequenced (University of California, Davis Sequencing Facility using BigDye Terminator v. 3.1 Cycle Sequencing Kit on an ABI Prism 3730 Genetic Analyzer). Positive control standard curves were generated by serial plasmid dilutions ranging from 1 to 1,00,000 plasmid copies/3ul (corresponding to 1.16E-9 − 1.16E-4 ng DNA) of the HRSV and HMPV controls.

Serial dilutions of known concentrations of HRSV and HMPV viral RNA, as determined by spectrophotometer, were spiked into 200 mg aliquots of verified HRSV- and HMPV-negative western lowland gorilla (*Gorilla gorilla gorilla*) feces from two healthy individuals housed at the San Francisco Zoo, an adult female and juvenile male. Approximately 25 g of feces was obtained from each gorilla by zookeepers; five grams of feces was stored in 35 ml of Ambion RNA*later*^®^ (Life Technologies, Carlsbad, CA) in five separate aliquots. The 10 samples (five from each gorilla) were then frozen at -80C after 24 h of refrigeration prior to laboratory testing. RNA was then extracted from feces (EURx GeneMATRIX Stool DNA Purification Kit CHIMERx, Milwaukee, WI) modified to simultaneously also extract carrier RNA (Köndgen et al. 2010) and cDNA transcribed (Invitrogen SuperScript III First-Strand Synthesis System). The range of spiked HRSV RNA was 6.8–6.8E-4 ng RNA, and the range for HMPV RNA was 623–6.23E-3 ng RNA; spiking amounts varied because of differing starting concentrations. All viral RNA dilutions were spiked in duplicate and run on real-time PCR assays in duplicate, along with their respective plasmid PC standard curves. PCR results were assessed both by Ct value and visualization by gel electrophoresis. Using the plasmid standard curve for relative quantitation, the amount of cDNA template for each spiking was extrapolated. DNA from PC plasmid dilutions and cDNA from spiking dilutions were further tested by conventional PCR for both viruses to amplify gene fragments for subsequent phylogenetic analysis.

### Outbreak Sample Collection

From May 2012 to March 2013 mountain gorillas in Rwanda, Uganda, and the Democratic Republic of Congo were monitored daily for signs of respiratory illness (RI), defined by veterinarians as at minimum nasal discharge, sneezing, and intermittent coughing (Spelman et al. 2013). Twenty fecal samples, representing 14 individual mountain gorillas exhibiting signs of respiratory illness, were collected from three gorilla groups experiencing RI outbreaks in Rwanda in June 2012 (Umubano gorilla group), August–October 2012 (Agashya group), and January–February 2013 (Sabyinyo group), and from two gorilla groups with individuals exhibiting signs of RI in June 2012. Fecal samples were collected by veterinarians and trackers who were able to identify individual gorillas and had been trained in fecal collection and daily animal health assessment. An outbreak was defined as clinically observable RI involving at least one third of the gorillas in a family group (minimum of 33% morbidity) for two or more consecutive days (Spelman et al. 2013). When possible, fecal samples were collected from any animal showing signs of RI on the first day with clinical signs and every other day for the duration of the animal’s illness. For all sampled gorillas, 3–5 g of feces were placed in 5–10 × volumes of Ambion RNA*later*^®^ Solution (Life Technologies, Carlsbad, CA), refrigerated for 24 h to allow adequate penetration of the sample by the storage solution, and then frozen at -80C in Rwanda until transported in liquid nitrogen to the UC Davis laboratory where they were stored at -80C until thawed for analysis.

### Fecal Sample Analysis

RNA was extracted from mountain gorilla fecal samples and cDNA transcribed as described for western lowland gorilla feces above. Comparison samples from healthy mountain gorillas without signs of RI were randomly selected from among fecal samples collected from habituated animals during the 2010 Virunga population census (*n* = 80) and from a gorilla in the Amohoro group that exhibited diarrhea but did not have RI in January 2013. To confirm the specificity of the real-time PCR assays with potential unknown viral genotypes, samples were tested using a conventional PCR protocol with the real-time PCR primers in the same concentrations but omitting the probe. Reaction conditions for both HRSV and HMPV were as follows: 94°C for 2 min, 45 cycles of 94°C for 20 s, 60°C for 30 s, and 72°C for 1 min, with a final extension at 72°C for 10 min. Samples that tested positive by real-time PCR were tested with the conventional PCR assays to amplify the larger G gene fragment for phylogenetic analysis. PCR products were visualized on 1.5% agarose gels and cloned and sequenced by Sanger sequencing.

A 490 bp sequence of the HRSV G gene, spanning the second hypervariable region, was used for phylogenetic analysis (Sato et al. 2005). Reference RSV-A sequences were downloaded from the GenBank database to represent each HRSV-A genotype (Pretorius et al. 2013), as well as strains that have recently been circulating globally over the past few years (Collins et al. 1987; Peret et al. 1998, 2000; Venter et al. 2001; Moura et al. 2004; Shobugawa et al. 2009; Agoti et al. 2012; Eshaghi et al. 2012; Lee et al. 2012; Choudhary et al. 2013; Khor et al. 2013; Auksornkitti et al. 2014). Nucleotide sequences were aligned with MUSCLE (Edgar 2004) in Geneious 7.0.6 (Biomatters, New Zealand) and trimmed to avoid large end gaps. Using Akaike’s Information Criterion in jModelTest 2.1.6 v20140903 (Darriba et al. 2012), the general time-reversible (GTR) evolutionary model was chosen, and maximum likelihood trees were constructed in Geneious with statistical significance of tree topology assessed by bootstrapping using 1,000 replicates.

## Results

### Laboratory Assay Detection Limit

In spiked gorilla feces, the HMPV real-time PCR assay detected 0.031 ng viral RNA/g spiked feces (6.23E-3 ng total RNA spiked), and the HRSV real-time PCR assay detected 0.085 ng viral RNA/g spiked feces (1.7E-2 ng total RNA spiked). Based on the PC standard curve, this corresponded to an average amount of 1.11E-9 ng cDNA template for HRSV and 2.45E-9 ng cDNA template for HMPV.

### Respiratory Cases and Outbreaks

Twenty fecal samples were collected from wild human-habituated mountain gorillas with signs of respiratory illness in Rwanda. The 20 fecal samples collected from five gorilla groups experiencing RI events included 10 individuals sampled once and four animals sampled 2–3× on separate days of their illness (Table 1 provides details for the 17 samples collected during the three group-wide outbreaks described below).

**Table 1:** Summary of Respiratory Outbreaks and Associated Morbidity in Three Mountain Gorilla Groups in Rwanda’s Volcanoes National Park, June 2012–February 2013, and Human Respiratory Syncytial Virus (HRSV) Testing Results of Fecal Samples from Clinically Ill Mountain Gorillas.

Outbreak dates (duration in days)	Umubano 12–17 Jun 2012 (6)	Agashya 30 Aug–4 Oct 2012 (36)	Sabyinyo 22 Jan–14 Feb 2013 (24)
Overall morbidity^1^ (%)	4/12 (33.3)	14/24 (58.3)	13/15 (86.7)
Adults > 8 yrs	2/7 (28.6)	6/10 (60)	7/8 (87.5)
Juveniles 3.5–8 years	2/4 (50)	7/12 (58.3)	3/4 (75)
Infants up to 3.5 years	0/1 (0)	1/2 (50)	3/3 (100)
Males	4/9 (44.4)	7/9 (77.8)	7/8 (87.5)
Females	0/3 (0)	7/15 (46.7)	6/7 (85.7)
Interventions^2^	0	0	2
HMPV-positive fecal samples	0/0 (0)	0/0 (0)	0/0 (0)
HRSV-positive fecal samples	0/2 (0)	6/6 (100)^3^	5/9 (55.5)^4^
HRSV-positive individuals	0/2 (0)	3/3 (100)	4/7 (57.1)

Data from group−wide outbreaks in three gorilla groups; samples (*n* = 3) were also collected from other small events from two additional gorilla groups (data not shown).

^1^Overall morbidity was defined as the number of animals that showed clinical signs divided by gorilla demographic group size during the outbreak; total numbers of subgroups did not always equal total group size, as age or sex was not known for all individuals.

^2^Interventions involved remotely administering (darting) gorillas with an antibiotic (ceftriaxone) and a non-steroidal anti-inflammatory (ketoprofen).

^3^These six samples were collected from three individuals, including the silverback, from which HRSV-positive fecal samples were collected on the 9/4, 9/18, and 9/20/12; an adult female, from which HRSV-positive fecal samples were collected on 9/20/12 and 10/2/12; and a juvenile male from a single collection on 9/20/12.

^4^These five samples were collected from four individuals, including the silverback, from which HRSV-negative fecal samples were collected on 2/2 and 2/6/13; an adult female, from which HRSV-positive fecal samples were collected on 1/22 and 1/28/13; and two adult females from which HRSV-positive fecal samples were collected on single occasions, 1/29/13 and 6/2/13.

**Figure 1:**
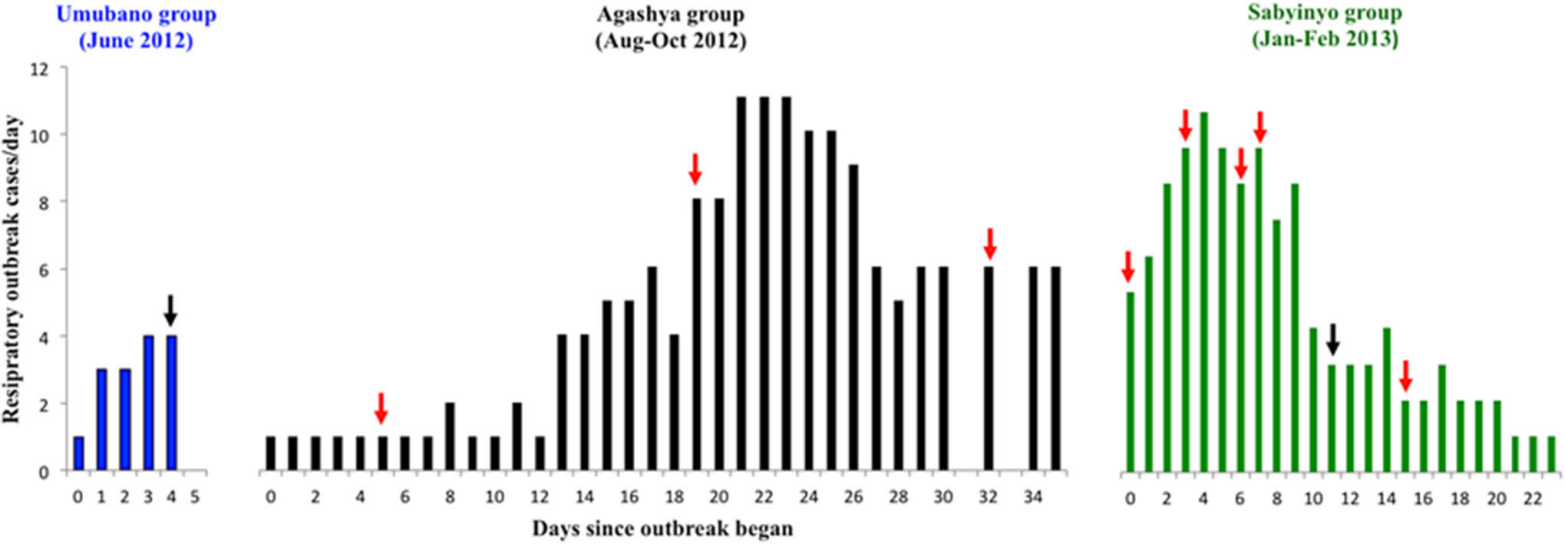
Clinical cases of respiratory illness in mountain gorillas during three outbreaks between June 2012 and February 2013 in Volcanoes National Park, Rwanda. Arrows represent the day on which fecal samples were opportunistically collected from one or more individuals during each outbreak (note that fecal samples were collected from some individuals on multiple, non-consecutive days over the course of an outbreak); red arrows indicate days on which at least one RSV-positive fecal sample was collected, while black arrows indicate days on which only RSV-negative fecal samples were collected. 12 RSV PCR-positives were detected, and six of these were confirmed by sequencing.

The outbreak affecting the Umubano group was the shortest in duration (6 days) and resulted in the lowest overall morbidity (33%) and the least severe clinical signs, just meeting the minimum criteria for the definition of RI including intermittent coughing, sneezing, and minimal nasal discharge. The outbreaks affecting Agashya and Sabyinyo groups were more severe and longer in duration, 36 and 24 days, respectively, with higher morbidities (58.3 and 86.7%; Fig. 1). No females or infants in the Umubano group developed signs of RI, whereas both males and females in Agashya and Sabyinyo groups of all age categories showed signs of RI. The Agashya and Sabyinyo group outbreaks affected individual gorillas more severely, as observed clinical signs included a productive cough, lethargy, poor appetite, elevated respiratory rate, and shallow breathing. Illness was severe enough to prompt veterinary medical interventions in two animals in the Sabyinyo group (an intramuscular administration of an antibiotic and anti-inflammatory drug via remote darting).

### Fecal Testing from Respiratory Illness Cases

None of the comparison fecal samples (*n* = 81) tested positive for HMPV or HRSV by either PCR assay. Samples obtained from the gorillas in the Umubano group exhibiting mild RI were also negative. All samples collected from the Agashya and Sabyinyo groups during their RI outbreaks tested negative for HMPV. However, multiple samples from both groups were positive for HRSV using the real-time PCR assay with small peaks at 43–45 cycles that were not visible upon gel electrophoresis of the PCR product. All samples were re-tested using the primers from the real-time assay in a conventional PCR assay. Twelve of 20 fecal samples from respiratory cases were positive for the HRSV N gene, and all comparison samples were negative. Eleven of the positive cases were detected in the Agashya and Sabinyo group-wide outbreaks (Table 1). The twelfth positive sample was from one gorilla in Group 11 and represented an isolated (non-outbreak related) case of respiratory illness. Three individuals from the Agashya group and four individuals from the Sabinyo group, exhibiting minimal to severe clinical signs, were HRSV-positive. One individual gorilla, an adult male from the Agashya group, was HRSV-positive over a 17-day period based on test results from three separate fecal samples (Fig. 1).

**Figure 2:**
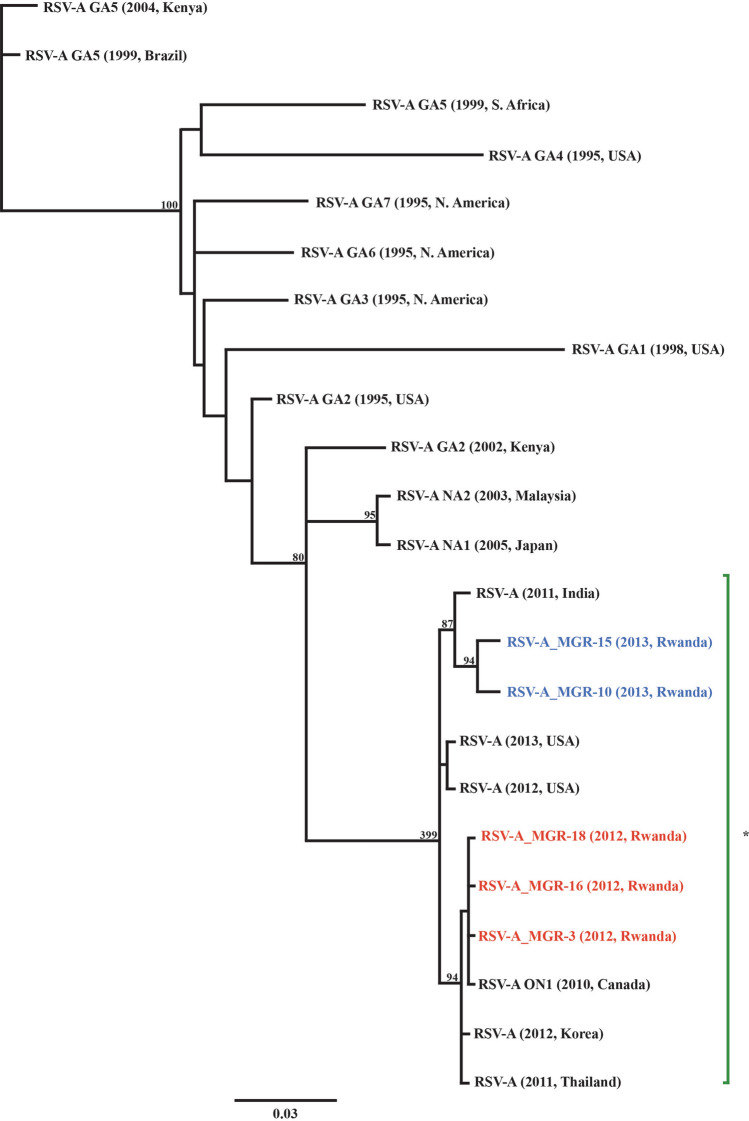
Maximum likelihood phylogenetic tree of a 490 bp fragment of the G gene showing the relationship between HRSV sequences amplified from mountain gorillas compared to known and recently circulating genotypes from people worldwide. Sequences in red are from mountain gorillas in Agashya group, while those in blue are from Sabyinyo group. Reference strains from GenBank (all from humans) include HRSV-A genotypes SAA1, ON1, NA1-2, and GA1-7, as well as isolates from 2011–2013; all have the year and country of collection noted. The clade marked by a green asterisk represents all available sample and reference strains with the 72-nucleotide gene duplication characteristic of the ON1 subtype. Bootstrap values of ≥ 70 are shown, and the bar indicates 0.03 nucleotide substitutions. Reference names and GenBank accession numbers included: 2011, Thailand (KC342446); 2012, Korea (JX627336); Strain ON1, 2010, Canada (JN257693); 2012, USA (KJ672440); 2013, USA (KM042388); 2011, India (KC731482); Strain NA1, 2005, Japan (AB470479); Strain NA2, 2003, Malaysia (JX256960); Strain GA1, 1987, USA (M74568); Strain GA2, 2002, Kenya (JQ838301); Strain GA2, 1995, USA (AF065258); Strain GA3, 1995, N. America (AF233920); Strain GA4, 1995, USA (AF065254); Strain GA5, 1999, Brazil (AY472094); Strain GA5, 2004, Kenya (JQ838418); Strain GA6, 1995, N. America (AF233918); Strain GA7; 1995, N. America (AF233904); Strain SAA1, 1999, S. Africa (AF348808).

Six samples positive by conventional PCR for the G gene were confirmed by sequencing. Upon maximum likelihood phylogenetic analysis, all sequences amplified from mountain gorilla feces were identified as subtype HRSV-A (ICTV reference accession M74568; Fig. 2). Of the six HRSV-A sequences amplified, five were unique sequences, all had ≥ 97.2% nucleotide similarity to each other and were most closely related to the ON1 genotype (Figs. 1, 2), a simultaneously emerging genotype circulating among humans worldwide (Song et al. 2017). Maximum Likelihood phylogenetic analysis of sequences detected in the Sabyinyo (Fig. 2: blue) and Agashya (Fig. 2: red) outbreaks clustered in subclades together; the sequences detected in the Sabyinyo group shared 99.4% nucleotide similarity with the ON1 genotype, while the sequences from the Agashya group shared 99.6% nucleotide similarity and were 100% identical to the ON1 human RSV reference genotype.

## Discussion

We tested fecal samples collected from wild human-habituated gorillas during two respiratory outbreaks and confirmed that clinically ill mountain gorillas were shedding human respiratory syncytial virus in their feces. Because no samples from clinically healthy gorillas had detectable HRSV, nor did the sample from the gorillas with diarrhea but no RI in other groups, our findings strongly suggested that HRSV-associated respiratory illness occurs in mountain gorillas and is associated with outbreaks. Our findings further corroborated the concern for the transmission of zoonotic pathogens between endangered great ape populations and people, particularly in settings where people are in frequent close contact with gorillas.

Experimental studies have shown that both HRSV and HMPV cause disease in captive non-human primates that is similar to clinical manifestation of viral infection in people, ranging from mild upper RI to pneumonia (van den Hoogen et al. 2001). Infection with and sharing of respiratory pathogens with people, specifically HRSV, has also been recently documented in western lowland gorillas and bonobos (Grützmacher et al. 2016, 2018). However, little was known about the attributable clinical signs in mountain gorillas, with the exception of a previous outbreak of severe RI leading to the deaths of two mountain gorillas that were infected with HMPV (Palacios et al. 2011). In people, clinical signs of HMPV and HRSV can range from mild to severe, and exposure to both viruses can result in reinfections throughout life with particularly severe cases in the very young (< 5 years of age), elderly (> 65 years of age), and immunocompromised (Falsey et al. 2003), as well as mild signs such as rhinorrhea and sore throat in otherwise healthy adults (Falsey and Walsh 2000).

Two main subtypes of HRSV have been described, HRSV-A and -B, based on antigenic and genetic variability (Anderson et al. 1985; Mufson et al. 1985). Most epidemiological phylogenetic studies have been based on the variable G gene sequence (Martinelli et al. 2014), as used in this study. Within both HRSV groups, there are multiple genotypes, often named for the country in which the sample was collected, even though HRSV viruses tend to vary seasonally rather than geographically. For example, the HRSV-A ON1 genotype, first identified in Ontario, Canada and characterized by a 72-nucleotide gene duplication in the amplified G gene region (Eshaghi et al. 2012), has spread globally and been reported in the US, India, Thailand, and Italy (Choudhary et al. 2013; Auksornkitti et al. 2014; Pierangeli et al. 2014). The HRSV strain detected in mountain gorilla fecal samples in this study was nearly genetically identical to the ON1 genotype and had the same 72-nt insertion. This finding suggests that this recently emerging virus genotype had been introduced into mountain gorillas from humans. However, it is not possible to speculate on the geographic origin of the strain detected in the mountain gorillas, as this genotype is now found worldwide and little is known about locally circulating genotypes due to limited testing for HRSV in the region.

The two outbreaks involving HRSV-positive samples were temporally separated by approximately 3.5 months, with the outbreak in Agashya group ending on 4 October 2012 and the outbreak in Sabyinyo group beginning on 22 January 2013. This timing, and what is known about HRSV shedding in people, supports the likelihood that these events were the result of separate introductions from humans to gorillas, rather than the spread of the virus from one gorilla group to the other. Although the two groups have adjacent home ranges, gorilla groups do not have frequent, sustained close contact with each other, and there were no reported conflicts or changes in group structure around the time of the outbreaks (such as an individual from Agashya group emigrating to Sabyinyo group). Also, the Agashya group outbreak ended more than two months before the beginning of the Sabyinyo group outbreak, and the maximum reported viral shedding period for HRSV in people is 17 days in adults (Hall et al. 2001) and up to 21 days in severely ill infants (Falsey and Walsh 2000).

HRSV can be transmitted by large droplets and contaminated fomites, on which the virus can remain infective for hours (Hall and Douglas 1981); thus, it is unlikely that this virus would remain intact for months in the environment. Also, based on our testing of gorilla fecal samples, there was no evidence for asymptomatic HRSV infections in gorillas as samples from comparison samples were all negative, and only humans are thought to be the reservoir for the virus (Dudas and Karron 1998). Interestingly, one outbreak associated with HRSV shedding began around the time of the rainy season and one in what is typically the dry season in Rwanda. Increases in respiratory illness in mountain gorillas in the rainy seasons have been previously reported (Watts 1998), while in chimpanzees, increased rates of respiratory illness were reported during the dry season (Lonsdorf et al. 2011). Given that the samples in this study are from only two outbreaks, we cannot draw conclusions about the likelihood of RI outbreaks associated with any particular season, but these data may indicate that this virus can infect gorillas throughout the year.

Our findings suggested that pathogenic respiratory viruses were repeatedly transmitted from people to mountain gorillas. Mountain gorilla tourists are often in closer proximity to the gorillas than the recommended distance (2.7 m on average, compared to the rule of a minimum 7 m) (Sandbrook and Semple 2006). Additionally, people who live in the communities surrounding the parks where mountain gorillas live self-report a high prevalence of symptoms such as coughing (72.1%) and fever (56.1%) (Guerrera et al. 2003). Though park personnel, researchers, and tourists are requested to refrain from visiting the mountain gorillas if they feel ill (Cranfield and Minnis 2007) and compliance appears to be improving due in part to strong educational efforts, self-identification and restraint can be difficult to enforce, particularly when tourists have limited time to visit the animals and have traveled to the area at great expense; furthermore, local people enter the park without the benefit of the health educational program (Hanes et al. 2018). Compounding this problem, a portion of infected and contagious people with viruses, such as HRSV, could be asymptomatic (Hall et al. 2001) and thus not aware that they could be the source of viruses transmissible to endangered great apes. These challenges, and the documentation of repeated introductions of human viruses to separate mountain gorilla family groups, suggest that additional biosafety protocols and the continued encouragement of adherence to existing protocols could reduce morbidity and mortality due to respiratory illness in mountain gorillas. Requiring people who are in close proximity to mountain gorillas to wear a surgical-grade facemask is widely recommended (Gilardi et al. 2015), and previous research has shown that both surgical and non-fitted P2 masks aid in reducing the risk of transmission of respiratory viruses (Jefferson et al. 2009; Loeb et al. 2009; MacIntyre et al. 2009). Maintenance of a safe distance between people and gorillas should also be enforced more stringently to aid in the prevention of contact with infected respiratory droplets from both symptomatic and asymptomatic individuals, since enforcement of visitation only by people not shedding human pathogens is almost impossible to enforce.

The COVID-19 pandemic has highlighted the importance of protecting great ape populations during human disease outbreaks. While there is no evidence at the time of writing that SARS-CoV-2 (the causative agent of COVID-19 disease) has infected wild great apes, transmission of human coronaviruses to wild great ape populations has the potential to cause severe respiratory disease and adverse health outcomes, posing a significant conservation threat (Patrono et al. 2018; Gillespie and Leendertz 2020). Given the evidence presented here of repeated virus transmission events and the impact of respiratory illness in human and gorillas, improved education and more holistic One Health approaches to improving the health of the living systems, including the health and livelihoods of people living and working near mountain gorillas, around the Virunga Massif may be the only solution. For example, in Rwanda, Gorilla Doctors initiated a preventive health program for park personnel who had regular contact with gorillas in an attempt to secure direct benefits for them from gorilla protection, as well as to improve community health and thus reduce the threat of infectious disease transmission from local people to the gorillas (Ali et al. 2004). Such programs are highly recommended (Gilardi et al 2015) and should be rigorously evaluated for their improvement in awareness of health risks to gorillas and their mitigation, as well as to encourage transparency about potential illnesses in visitors to gorillas and their ecosystems.

## References

[CR1] Agoti CN, Mwihuri AG, Sande CJ, Onyango CO, Medley GF, Cane PA, Nokes DJ (2012). Genetic relatedness of infecting and reinfecting respiratory syncytial virus strains identified in a birth cohort from rural Kenya. Journal of Infectious Diseases.

[CR2] Ali R, Cranfield M, Gaffikin L, Mudakikwa A, Ngeruka L, Whittier C (2004). Occupational Health and Gorilla Conservation in Rwanda. International Journal of Occupational; and Environmental Health.

[CR3] Alvarez KADA, Vollm J (2004). Zoonotic diseases shared by gorillas and humans: a veterinary perspective. Gorilla Journal.

[CR4] Anderson LJ, Hierholzer JC, Tsou C, Hendry RM, Fernie BF, Stone Y, McIntosh K (1985). Antigenic characterization of respiratory syncytial virus strains with monoclonal antibodies. Journal of Infectious Diseases.

[CR5] Aslanzadeh J, and Tang Y-W, 2011. Human Metapneumovirus. In: *Molecular Detection of Human Viral Pathogens*, Dongyou L (editor), Boca Raton, FL: CRC Press, pp 479–486.

[CR6] Auksornkitti V, Kamprasert N, Thongkomplew S, Suwannakarn K, Theamboonlers A, Samransamruajkij R, Poovorawan Y (2014). Molecular characterization of human respiratory syncytial virus, 2010–2011: identification of genotype ON1 and a new subgroup B genotype in Thailand. Archives of Virology.

[CR7] Buitendijk H, Fagrouch Z, Niphuis H, Bogers WM, Warren KS, Verschoour EJ (2014). Retrospective serology study of respiratory virus infections in captive great apes. Viruses.

[CR8] Chi F, Leider M, Leendertz F, Bergmann C, Boesch C, Schenk S, Pauli G, Ellerbrok H, Hakenbeck R (2007). New *Streptococcus pneumoniae* clones in deceased wild chimpanzees. Journal of Bacteriology.

[CR9] Choudhary ML, Wadhwa BS, Jadhav SM, and Chadha MS, 2013. Complete genome sequences of two human respiratory syncytial virus genotype A strains from India, RSV-A/NIV1114046/11 and RSV-A/NIV1114073/11. *Genome Announcements* **1**. 10.1128/genomeA.00165-13 PMC3735064 23887906

[CR10] Collins PL, Olmsted RA, Spriggs MK, Johnson PR, Buckler-White AJ (1987). Gene overlap and site-specific attenuation of transcription of the viral polymerase L gene of human respiratory syncytial virus. Proceedings of the National Academy of Sciences.

[CR11] Cranfield M, Minnis R (2007). An integrated health approach to the conservation of Mountain gorillas Gorilla beringei beringei. International Zoo Yearbook.

[CR12] Cranfield MR (2008). Mountain gorilla research: the risk of disease transmission relative to the benefit from the perspective of ecosystem health. American Journal of Primatology.

[CR13] Darriba D, Taboada G, Doallo R, Posada D (2012). jModelTest 2: more models, new heuristics and parallel computing. Nature Methods.

[CR14] Dudas RA, Karron RA (1998). Respiratory Syncytial Virus Vaccines. Clinical Microbiology Reviews.

[CR15] Edgar RC (2004). MUSCLE: multiple sequence alignment with high accuracy and high throughput. Nucleic Acids Research.

[CR16] Eshaghi A, Duvvuri VR, Lai R, Nadarajah JT, Li A, Patel SN, Low DE, Gubbay JB (2012). Genetic variability of human respiratory syncytial virus A strains circulating in Ontario: A novel genotype with a 72 nucleotide G gene duplication. PLoS ONE.

[CR17] Falsey AR, Erdman D, Anderson LJ, Walsh EE (2003). Human metapneumovirus infections in young and elderly adults. Journal of Infectious Diseases.

[CR18] Falsey AR, Walsh EE (2000). Respiratory syncytial virus infection in adults. Clinical Microbioly Review.

[CR19] Gilardi KV, Gillespie TR, Leendertz FH, Macfie EJ, Travis DA, Whittier CA, Williamson EA (2015). Best Practice Guidelines for Health Monitoring and Disease Control in Great Ape Populations.

[CR20] Gillespie T, Leendertz F (2020). COVID-19: protect great ape populations during human pandemics. Nature.

[CR21] Grützmacher KS, Keil V, Metzger S, Wittiger L, Herbinger I, Calvignac-Spencer S, Mätz-Rensing K, Haggis O, Savary L, Köndgen S, Leendertz FH (2018). Human Respiratory Syncytial Virus and *Streptococcus pneumoniae* Infection in Wild Bonobos. EcoHealth.

[CR22] Grützmacher KS, Köndgen S, Keil V, Todd A, Feistner A, Herbinger I, Petrzelkova K, Fuh T, Leendertz SA, Calvignac-Spencer S, Leendeertz FH, 2016. Co-detection of respiratory syncytial virus in habituated wild western lowland gorillas and humans during a respiratory disease outbreak. *EcoHealth*: DOI: 10.1007/s10393-016-1144-6. 10.1007/s10393-016-1144-6 PMC7088376 27436109

[CR23] Guerrera W, Sleeman JM, Jasper SB, Pace LB, Ichinose TY, Reif JS (2003). Medical Survey of the Local Human Population to Determine Possible Health Risks to the Mountain Gorillas of Bwindi Impenetrable Forest National Park, Uganda. International Journal of Primatology.

[CR24] Hall CB, Douglas RG (1981). Modes of transmission of respiratory syncytial virus. The Journal of Pediatrics.

[CR25] Hall CB, Long CE, Schnabel KC (2001). Respiratory syncytial virus infections in previously healthy working adults. Clinical Infectious Diseases.

[CR26] Hanes AC, Kalema-Zikusoka G, Svensson MS, Hill CM (2018). Assessment of health risks posed by tourists visiting mountain gorillas in Bwindi Impenetrable National Park, Uganda. Primate Conservation.

[CR27] Hassell, JM, D Zimmerman, MR Cranfield, K Gilardi, A Mudakikwa, J Ramer, E Nyirakaragire, and LJ Lowenstine, 2017. Morbidity and morality in infant mountain gorillas (*Gorilla beringei beringei*): A 46-year retrospective. *American Journal of Primatology* **79**(100): DOI: 10.1002/ajp.22686. 10.1002/ajp.22686 28749595

[CR28] Hickey JR, Basabose A, Gilardi KV, Greer D, Nampindo, S, Robbins, MM, and Stoinski, TS, 2018a. *Gorilla beringei ssp. beringei*. The IUCN Red List of Threatened Species 2018: e.T39999A17989719. 10.2305/IUCN.UK.2018-2.RLTS.T39999A17989719.en.

[CR29] Hickey J, Granjon AC, Vigilant L, Eckardt W, Gilardi VK, Cranfield M, Musana A, Masozera AB, Babaasa D, Ruzigandekew F, and Robbins MM, 2018b. *Virunga 2015-2016 Surveys: Monitoring Mountain Gorillas, Other Selected Mammals, and Illegal Activities.* Final Report. IGCP and GVTC, Kigali, Rwanda.

[CR30] Hickey JR, Uzabaho E, Akantorana M, Arinaitwe J, Bakebwa I, Bitariho R, Eckardt W, Gilardi KV, Katutu J, Kayijamahe C, Kierepka EM, Mugabukomeye B, Musema A, Mutabaazi H, Robbins MM, Sacks BN, and Zikusoka, GK, 2019. Bwindi- Sarambwe 2018 Surveys: monitoring mountain gorillas, other select mammals, and human activities. GVTC, IGCP and partners, Kampala, Uganda, 40p.

[CR31] Homsy J, 1999. *Ape tourism and human diseases: how close should we get?* Report of a Consultancy for the International Gorilla Conservation Programme. (http://www.igcp.org/pdf/homsy_rev.pdf)

[CR32] Jefferson T, Del Mar C, Dooley L, Ferroni E, Al-Ansary LA, Bawazeer GA, van Driel ML, Foxlee R, Rivetti A (2009). Physical interventions to interrupt or reduce the spread of respiratory viruses: systematic review. BMJ.

[CR33] Kalpers J, Williamson EA, Robbins MM, McNeilage A, Nsamurambaho A, Lola N, Mugiri G (2003). Gorillas in the crossfire: population dynamics of the Virunga mountain gorillas over the past three decades. Oryx.

[CR34] Kaur T, Singh J, Tong S, Humphrey C, Clevenger D, Tan W, Szekely B, Wang Y, Li Y, Alex Muse E, Kiyono M, Hanamura S, Inoue E, Nakamura M, Huffman MA, Jiang B, Nishida T (2008). Descriptive epidemiology of fatal respiratory outbreaks and detection of a human-related metapneumovirus in wild chimpanzees (*Pan troglodytes*) at Mahale Mountains National Park, Western Tanzania. American Journal of Primatology.

[CR35] Khor C-S, Sam IC, Hooi P-S, Chan Y-F (2013). Displacement of predominant respiratory syncytial virus genotypes in Malaysia between 1989 and 2011. Infection, Genetics and Evolution.

[CR36] Kilbourn AM, Karesh WB, Wolfe ND, Bosi EJ, Cook RA, Andau M (2003). Health evaluation of free-ranging and semi-captive orangutans (*Pongo pygmaeus pygmaeus*) in Sabah, Malaysia. Journal of Wildlife Disease.

[CR37] Klemenc J, Ali SA, Johnson M, Tollefson SJ, Talbot HK, Hartert TV, Edwards KM, Williams JV (2012). Real-time reverse transcriptase PCR assay for improved detection of human metapneumovirus. Journal of Clinical Virology.

[CR38] Kooriyama T, Okamoto M, Yoshida T, Nishida T, Tsubota T, Saito A, Tomonaga M, Matsuzawa T, Akari H, Nishimura H, Miyabe-Nishiwaki T (2013). Epidemiological study of zoonoses derived from humans in captive chimpanzees. Primates.

[CR39] Köndgen S, Calvignac-Spencer S, Grützmacher K, Keil V, Matz-Rensing K, Nowak K, Metzger S, Kiyang J, Lubke Becker A, Deschner T, Wittig RM, Lankester F, Leendertz F (2017). Evidence for human *Streptococcus pneumoniae* in wild and captive chimpanzees: A potential threat to wild populations. Sci Rep.

[CR40] Köndgen S, Kuhl H, N'Goran PK, Walsh PD, Schenk S, Ernst N, Biek R, Formenty P, Matz-Rensing K, Schweiger B, Junglen S, Ellerbrok H, Nitsche A, Briese T, Lipkin WI, Pauli G, Boesch C, Leendertz F (2008). Pandemic human viruses cause decline of endangered great apes. Current Biology.

[CR41] Köndgen S, Schenk S, Pauli G, Boesch C, Leendertz F (2010). Noninvasive Monitoring of Respiratory Viruses in Wild Chimpanzees. Ecohealth.

[CR42] Lee W-J, Kim Y-j, Kim D-W, Lee HS, Lee HY, Kim K (2012). Complete genome sequence of human respiratory syncytial virus genotype A with a 72-nucleotide duplication in the attachment protein G gene. Journal of Virology.

[CR43] Liu D, 2011. Human Respiratory Syncytial Virus. In: *Molecular Detection of Human Viral Pathogens*, Liu D (editor), Boca Raton, FL: CRC Press, pp 497–503.

[CR44] Loeb M, Dafoe N, Mahony J, John M, Sarabia A, Glavin V, Webby R, Smieja M, Earn DJ, Chong S, Webb A, Walter SD (2009). Surgical mask vs n95 respirator for preventing influenza among health care workers: A randomized trial. JAMA.

[CR45] Lonsdorf E, Murray C, Lonsdorf E, Travis D, Gilby I, Chosy J, Goodall J, Pusey AE (2011). A retrospective analysis of factors correlated to chimpanzee (*Pan troglodytes schweinfurthii*) respiratory health at Gombe National Park, Tanzania. Ecohealth.

[CR46] MacIntyre CR, Cauchemez S, Dwyer DE, Seale H, Cheung P, Browne G, Fasher M, Wood J, Booy R, Ferguson N (2009). Face mask use and control of respiratory virus transmission in households. Emerging Infectious Diseases.

[CR47] Martinelli M, Frati ER, Zappa A, Ebranati E, Bianchi S, Pariani E, Amendola A, Zehender G, Tanzi E (2014). Phylogeny and population dynamics of respiratory syncytial virus (Rsv) A and B. Virus Research.

[CR48] Moura FEA, Blanc A, Frabasile S, Delfraro A, de Sierra MJ, Tome L, Ramos EA, Siqueira MM, Arbiza J (2004). Genetic diversity of respiratory syncytial virus isolated during an epidemic period from children of northeastern Brazil. Journal of Medical Virology.

[CR49] Mufson MA, Örvell C, Rafnar B, Norrby E (1985). Two Distinct Subtypes of Human Respiratory Syncytial Virus. Journal of General Virology.

[CR50] Negrey J, Reddy R, Scully E, Phillips-Garcia S, Owens L, Langergraber K, Mitani J, Thompson M, Wrangham R, Muller M, Otali E, Machanda Z, Hyeroba D, Grindle K, Pappas T, Palmenberg A, Gern J, Goldberg T (2019). Simultaneous outbreaks of respiratory disease in wild chimpanzees caused by distinct viruses of human origin. Emerging Microbes & Infections.

[CR51] Nizeyi J, Rwego I, Erume J, Kalema G, Cranfield M, Graczyk T (2001). Campylobacteriosis, salmonellosis, and shigellosis in free-ranging human-habituated mountain gorillas of Uganda. Journal of Wildlife Diseases.

[CR52] Nizeyi J, Sebunya D, Dasilva A, Cranfield M, Pieniazek N, Graczyk T (2002). Cryptosporidiosis in people sharing habitats with free-ranging mountain gorillas (Gorilla gorilla beringei), Uganda. American Journal of Tropical Medicine and Hygiene.

[CR53] Nolen RS (2006). Gorilla conservation project takes one-health approach. Journal of the American Veterinary Medical Association.

[CR54] Palacios G, Lowenstine LJ, Cranfield MR, Gilardi KVK, Spelman L, Lukasik-Braum M, Kinani JF, Mudakikwa A, Nyirakaragire E, Bussetti AV, Savji N, Hutchison S, Egholm M, Lipkin WI (2011). Human metapneumovirus infection in wild mountain gorillas, Rwanda. Emerging Infectious Diseases.

[CR55] Patrono LV, Samuni L, Corman VM, Nourifar L, Röthemeier C, Wittig RM, Drosten C, Calvignac-Spencer S, Leendertz FH (2018). Human coronavirus OC43 outbreak in wild chimpanzees, Côte d´Ivoire, 2016. Emerging Microbes & Infections.

[CR56] Peret TCT, Hall CB, Hammond GW, Piedra PA, Storch GA, Sullender WM, Tsou C, Anderson LJ (2000). Circulation patterns of group A and B human respiratory syncytial virus genotypes in 5 communities in North America. Journal of Infectious Diseases.

[CR57] Peret TCT, Hall CB, Schnabel KC, Golub JA, Anderson LJ (1998). Circulation patterns of genetically distinct group A and B strains of human respiratory syncytial virus in a community. Journal of General Virology.

[CR58] Pierangeli A, Trotta D, Scagnolari C, Ferreri M, Nicolai A, Midulla F, Marinelli K, Antonelli G, and Bagnarelli P, 2014. Rapid spread of the novel respiratory syncytial virus A ON1 genotype, central Italy, 2011 to 2013. *Euro surveillance: bulletin Européen sur les maladies transmissibles= European communicable disease bulletin* **19**. 10.2807/1560-7917.es2014.19.26.20843 25011065

[CR59] Piyaratna R, Tollefson SJ, Williams JV (2011). Genomic analysis of four human metapneumovirus prototypes. Virus Research.

[CR60] Pretorius MA, van Niekerk S, Tempia S, Moyes J, Cohen C, Madhi SA, Venter M, and SARU Surveillance Group (2013). Replacement and positive evolution of subtype A and B respiratory syncytial virus G- protein genotypes from 1997–2012 in South Africa. Journal of Infectious Diseases.

[CR61] Reiche J, Schweiger B (2009). Genetic variability of group A human respiratory syncytial virus strains circulating in Germany from 1998 to 2007. Journal of Clinical Microbiology.

[CR62] Robbins MM, Gray M, Fawcett K, Nutter FB, Uwingeli P, Mburanumwe I, Kagoda E, Basabose A, Stoinski TS, Cranfield M, Byamukama J, Spelman LH, Robbins AM (2011). Extreme conservation leads to recovery of the Virunga mountain gorillas. PLoS ONE.

[CR63] Ryan SJ, Walsh PD (2011). Consequences of non-intervention for infectious disease in African great apes. PLoS ONE.

[CR64] Sandbrook C, Semple S (2006). The rules and the reality of mountain gorilla *Gorilla beringei beringei* tracking: how close do tourists get?. Oryx.

[CR65] Sato M, Saito R, Sakai T, Sano Y, Nishikawa M, Sasaki A, Shobugawa Y, Gejyo F, Suzuki H (2005). Molecular epidemiology of respiratory syncytial virus infections among children with acute respiratory symptoms in a community over three seasons. Journal of Clinical Microbiology.

[CR66] Scully EJ, Basnet S, Wrangham RW, Muller MN, Otali E, Hyeroba D, Grindle KA, Pappas TE, Thompson ME, Machanda Z, Watters KE, Palmenberg AC, Gern JE, Goldberg TL (2018). Lethal Respiratory Disease Associated with Human Rhinovirus C in Wild Chimpanzees, Uganda, 2013. Emerging Infectious Diseases.

[CR67] Setchell JM, Fairet E, Shutt K, Waters S, Bell S (2016). Biosocial conservation: Integrating biological and ethnographic methods to study human-primate interactions. International journal of primatology.

[CR68] Shobugawa Y, Saito R, Sano Y, Zaraket H, Suzuki Y, Kumaki A, Dapat I, Oguma T, Yamaguchi M, Suzuki H (2009). Emerging genotypes of human respiratory syncytial virus subgroup A among patients in Japan. Journal of Clinical Microbiology.

[CR69] Slater O, Terio KA, Zhang Y, Erdman DD, Schneider E, Kuypers JM, Wolinksy SM, Kunstman KJ, Kunstman J, Kinsel MJ, Gamble KC (2014). Human metapneumovirus infection in chimpanzees. United States. Emerging Infectious Diseases.

[CR70] Song J, Zhang Y, Wang H, Shi J, Zhang Sun L, X, Yang Z, Guan W, Zhang H, Yu P, Xie Z, Cui A, Ng TI, and Xu W, (2017). Emergence of ON1 genotype of human respiratory syncytial virus subgroup A in China between 2011 and 2015. Nature Scientific Reports.

[CR71] Spelman LH, Gilardi KVK, Lukasik-Braum M, Kinani JF, Nyirakaragire E, Lowenstine LJ, Cranfield MR (2013). Respiratory disease in mountain gorillas (*Gorilla beringei beringei*) in Rwanda, 1990–2010: Outbreaks, clinical course, and medical management. Journal of Zoo and Wildlife Medicine.

[CR72] Szentiks CA, Köndgen S, Silinski S, Speck S, Leendertz FH (2009). Lethal pneumonia in a captive juvenile chimpanzee (*Pan troglodytes*) due to human-transmitted human respiratory syncytial virus (HRSV) and infection with *Streptococcus pneumoniae*. Journal of Medical Primatology.

[CR73] Unwin S, Chatterton J, Chantrey J (2013). Management of severe respiratory tract disease caused by human respiratory syncytial virus and Streptococcus pneumoniae in captive chimpanzees (*Pan troglodytes*). Journal of Zoo and Wildlife Medicine.

[CR74] van den Hoogen BG, de Jong JC, Groen J, Kuiken T, de Groot R, Fouchier RAM, Osterhaus AD (2001). A newly discovered human pneumovirus isolated from young children with respiratory tract disease. Nature Medicine.

[CR75] Venter M, Madhi SA, Tiemessen CT, Schoub BD (2001). Genetic diversity and molecular epidemiology of respiratory syncytial virus over four consecutive seasons in South Africa: identification of new subgroup A and B genotypes. Journal of General Virology.

[CR76] Watts DP (1998). Seasonality in the ecology and life histories of mountain gorillas (Gorilla gorilla beringei). International Journal of Primatology.

